# Endovascular management of life threatening bleeding from a radiation induced internal iliac artery branch pseudoaneurysm

**DOI:** 10.1186/s42155-019-0073-1

**Published:** 2019-08-27

**Authors:** Abhijit Salaskar, Philip Blumenfeld, Joseph Calandra, Michael Hamblin

**Affiliations:** 1 Department of Radiology, Amita Saint Francis Hospital, Evanston, IL 60202 USA; 2 Department of Radiation Oncology, Amita Saint Francis Hospital, Evanston, IL 60202 USA

**Keywords:** Pseudoaneurysm, Iliac artery pseudoaneurysm, Radiation therapy induced, Radiation induced

## Abstract

**Background:**

Among radiation induced arterial complications, stenoses and occlusions are commonly reported. Radiation induced pseudoaneurysms (PSA) and their management outcomes are rarely reported.

**Case presentation:**

A 48 year old male underwent low anterior resection surgery for a clinically staged T2N0M0 rectal adenocarcinoma and adjuvant chemoradiation for the findings of lymphovascular invasion and focally positive distal margin 2 years prior to current admission. The patient now presented with syncope and anemia. The patient was hypotensive after an episode of hematochezia during the hospital stay. An urgent sigmoidoscopy revealed bleeding from friable necrotic rectal mucosa with focal pulsations along the left posterolateral aspect of the rectal wall. An emergent pelvic angiogram revealed active extravasation from a 3 mm PSA from the anterior division of left internal iliac artery. After coil embolization of the affected vascular branch on either side of the neck of PSA, there was no opacification of PSA or extravasation. The patient remained asymptomatic for 3 years.

**Conclusions:**

Radiation induced PSA must be considered in the absence of trauma. Endovascular coil-embolization of radiation induced PSAs from small caliber vessels can be an effective treatment.

## Background

Among radiation induced vascular complications, arterial stenoses and occlusions are more commonly reported, however radiation induced pseudoaneurysms (PSA) are extremely rare and are scarcely reported (De Baere et al., 1998). Also there are no prior studies reporting the outcomes of endovascular embolization of radiation induced PSA.

## Case presentation

**Fig. 1:**
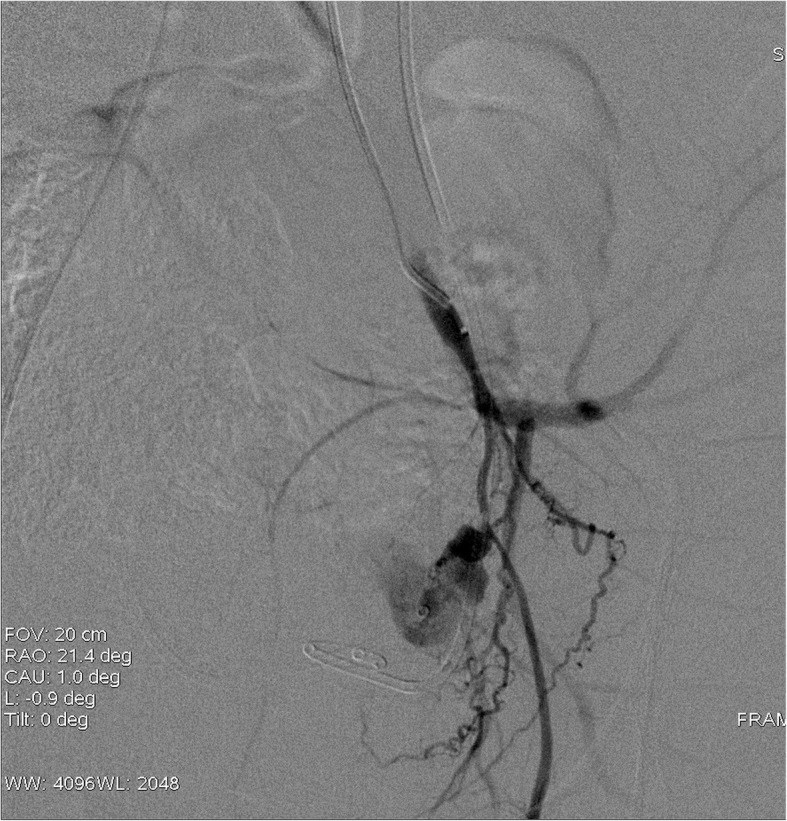
Selective angiography of LIIA demonstrated an extravasation from a 3 mm pseudoaneurysm from the branch of anterior division of left internal iliac artery

**Fig. 2:**
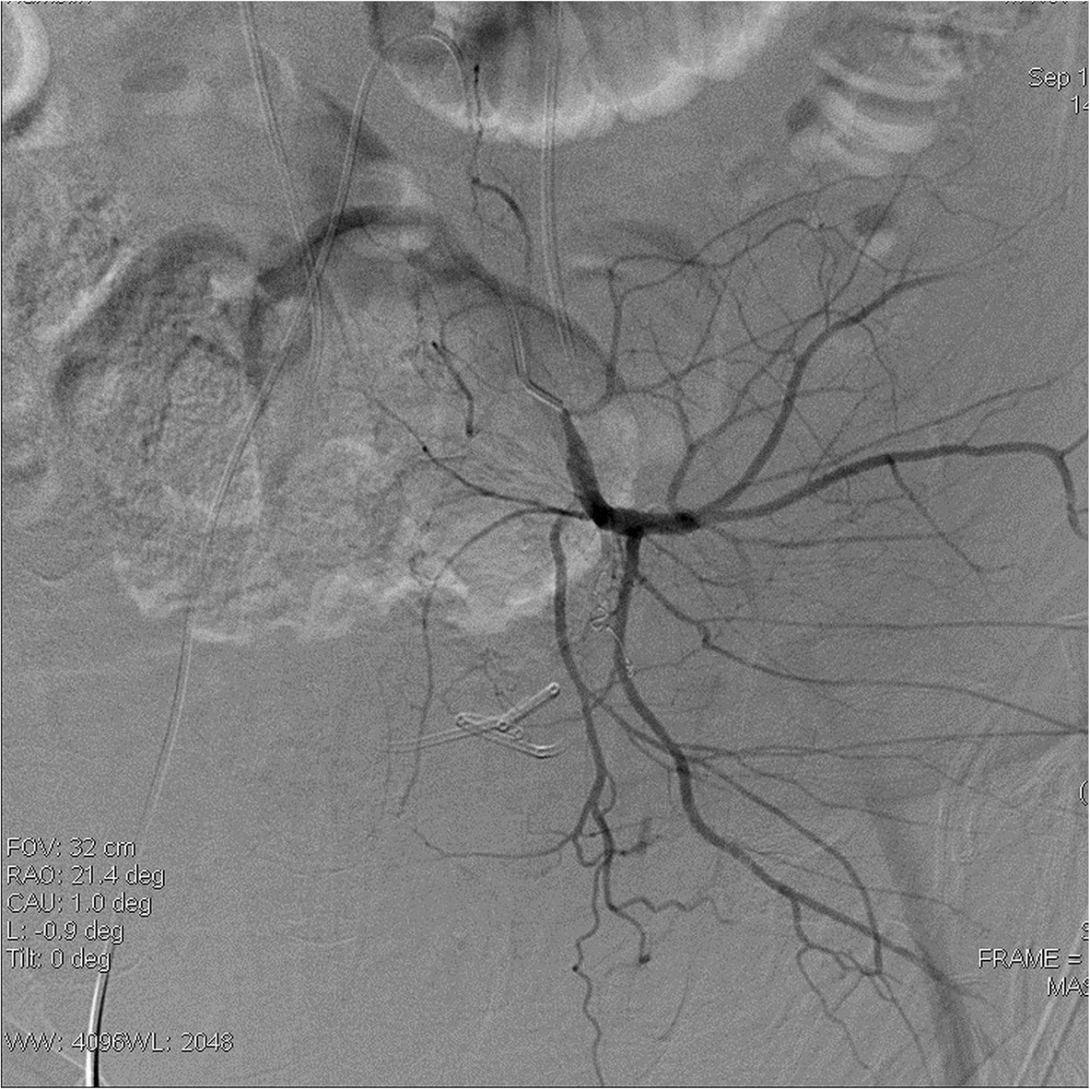
Angiography after coil embolization demonstrated non opacification of the pseudoaneurysm and absence of extravasation

Our patient is a 48 year old male who was diagnosed with early stage rectal cancer. The patient underwent an uneventful low anterior resection of rectal cancer due to initial clinical stage of T2N0M0. Pathological evaluation of the resected rectosigmoid specimen demonstrated pT2N0M0 rectal adenocarcinoma with lymphovascular invasion and focally positive distal resection margin. Due to these findings the patient received 4500 cGy of adjuvant radiation to the pelvis followed by a 900 cGy boost to the rectum and mesorectum. Patient also received concurrent chemotherapy including Capecitabine. Two years after the adjuvant chemoradition, patient presented to our institution with multiple episodes of syncope and anemia. During the hospital admission, the patient became hypotensive after an episode of hematochezia. An urgent sigmoidoscopy revealed bleeding from friable necrotic rectal mucosa with focal pulsations along the left posterolateral aspect of the rectal wall. The location of the anticipated bleeding vessel, along with history of prior surgery and radiotherapy made this patient a poor surgical candidate. We performed an emergent pelvic angiogram. The selective angiography of the left internal iliac artery (LIIA) showed persistent extravasation from a 3 mm PSA from the branch of an anterior division of LIIA (Fig. 1). In order to prevent the possible increase in the rupture and bleeding from the PSA sac, coil embolization of PSA sac was avoided. Instead a 3 mm × 2.5 mm fibered platinum pushable coils (Boston Scientific VortX 18) were initially deployed in the affected vascular branch distally and at the neck of the PSA to prevent retrograde bleeding from collaterals. Then two other 3 mm × 2.5 mm coils and gelfoam were deployed proximal toneck of the PSA. On subsequent angiography, the PSA did not opacify and there was no evidence of extravasation (Fig. 2). Three years after the embolization, patient has remained asymptomatic.

## Conclusion

Among radiation induced vascular complications, arterial stenoses and occlusions are more commonly reported than perforations or PSAs (De Baere et al., 1998). The most common causes of PSAs are iatrogenic or traumatic. However in our case, prior procedures such as low anterior resection surgery (2 years prior) and CT guided biopsy of the benign presacral mass (1 year prior) via right transgluteal approach were distant from the location of the LIIA branch PSA. The contrast enhanced CT which was done after the rectal surgery or after CT guided biopsy of the presacral mass did not show any PSA. Thus the possibility of this PSA being secondary to trauma was very less likely. Our patient underwent low anterior resection of rectal cancer due to initial clinical staging of T2N0M0. The pathological evaluation of the resected rectosigmoid specimen demonstrated focally positive distal surgical resection margin and lymphovascular invasion. There was no definite tumor invasion into the pericolorectal tissue obviating the possibility of primary cancer associated PSA. After embolization of PSA, the biopsy of necrotic rectal mucosa adjacent to the location of PSA was benign. This indicated that the PSA was not associated with the recurrence of the tumor.

**Fig. 3:**
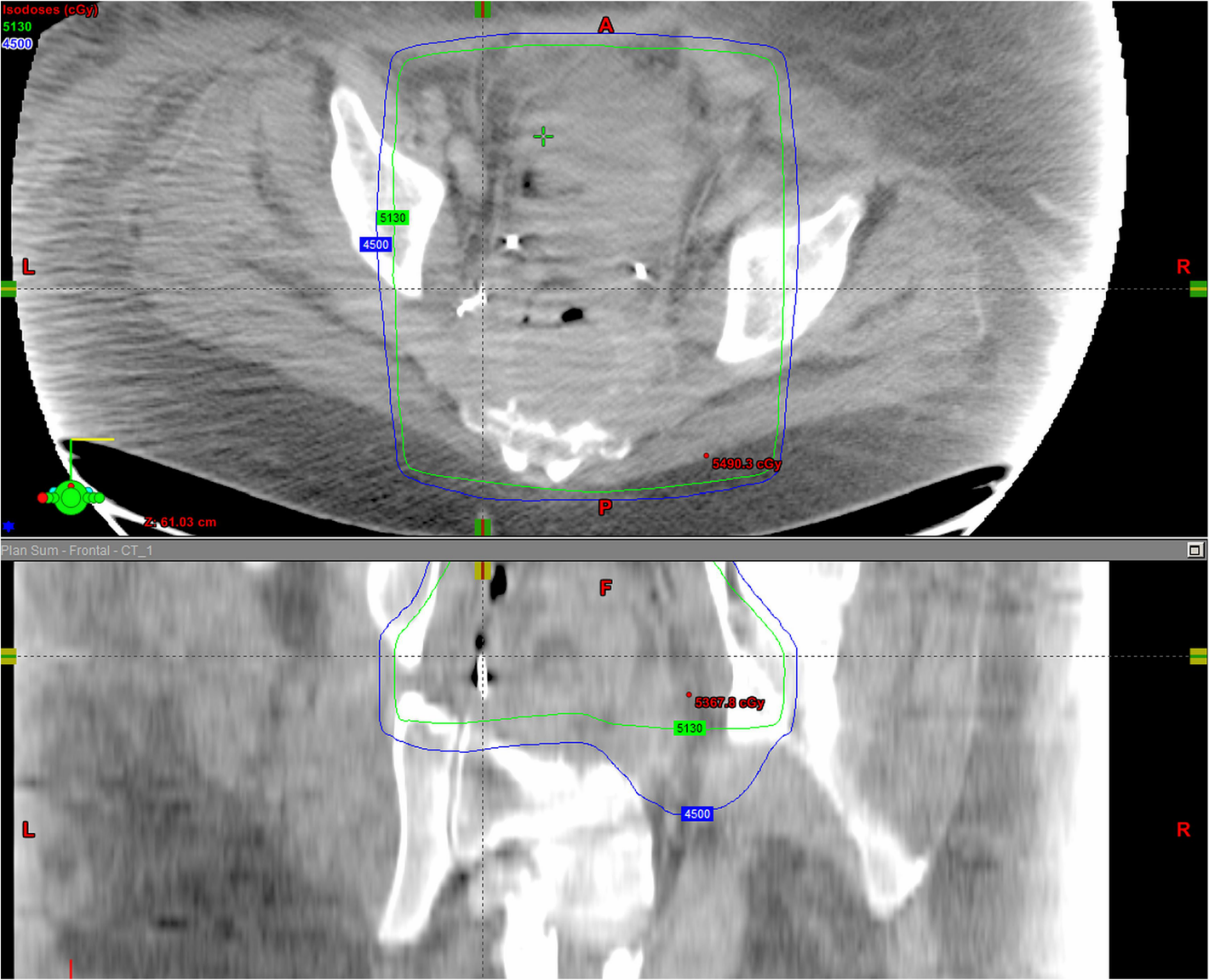
Radiation dose distribution images superimposed on CT angio abdomen and pelvis following PSA embolization. The PSA is noted to be within the 95% isodose line (5130 cGy, green line). Of note bilateral ureteral stents are seen anterior to the left pelvic embolization coils

There is paucity of data correlating radiation dose with vessel PSA formation. A prior case report (De Baere et al., 1998) demonstrated that four patients had common, internal or external iliac PSAs after receiving 3000–6500 cGy of radiation to pelvis (De Baere et al., 1998). Moreover, an animal study reported long term changes in the media and adventitia of the vessels after administration of 4000 R i.e. 3500 cGy of radiation dose to the canine femoral arteries (Fonkalsrud et al., 1977). In our case, the patient received 4500 cGy in 25 fractions to the pelvis followed by a 900 cGy in 5 fraction boost. In order to estimate radiotherapy dose to the area of the PSA, we recreated the radiation dose distribution images superimposed on the CT angio abdomen pelvis obtained after the embolization of LIIA branch PSA. The region surrounding the embolized LIIA branch PSA previously received approximately 95% of the total dose of 5400 cGy (5130 cGy, green isodose line seen on Fig. 3). This approximate dose is within the same range as the above studies.

Pre-operative radiotherapy is the preferred sequencing for patients with locally advanced rectal cancer compared to post-operative radiation. A prospective randomized trial has indicated that the adjuvant radiation therapy was associated with a higher incidence of radiation toxicity including anastomotic leakage and wound complications when compared to neoadjuvant radiation therapy. The study did not report or compare any radiation induced vascular complications (Sprenger et al., 2018). Of note, our patient did experience grade 3 proctitis and found to have friable rectal mucosa at the prior anastomotic site 2 years after the adjuvant radiation therapy requiring a bowel diversion.

Endovascular approach has been preferred over the open surgical approach in the management of iatrogenic PSAs as it obviates the need for surgical incision and wide dissection in the vicinity of the injured vessel. Also in patients with pelvic cancers, access to iliac vessels is often difficult due to prior surgery or radiation. In the prior case reports, the management of bleeding radiation induced pelvic PSAs included 10 mm covered stent placement in the common iliac artery PSA with only 9 month follow up, temporary balloon occlusion of a PSA arising from the iliac bifurcation with subsequent surgical ligation and axillo-femoral bypass, bare tungsten coil packing of an external iliac artery PSA with subsequent recurrent hemorrhage which was later treated by external iliac occlusion and femoro-femoral bypass (De Baere et al., 1998).

In our patient a 3 mm PSA was arising from an approximately 3 mm diameter branch of an anterior division of left internal iliac artery, most likely a middle rectal artery. Considering the small size and tortuosity of this affected end branch vessel, coil embolization to isolate the PSA sac was preferred. Coil embolization of PSA sac itself was avoided to prevent possible increase in rupture and bleeding from the PSA sac. For PSA arising from vessels with large caliber, covered stents can be used. Thus radiation induced PSA must be included in the differential diagnosis in the absence of trauma. Endovascular coil embolization of radiation induced PSAs arising from the small caliber pelvic vessels can serve as an effective treatment.

## Data Availability

Not applicable.

## Abbreviations

LIIA: Left internal iliac artery
PSA: Pseudoaneurysm
